# Differentiation of Typhoid and Para-Typhoid Bacilli

**Published:** 1916-03

**Authors:** 


**Differentiation of Typhoid and Para-Typhoid Bacilli.** Pierre Paul Levy and Pasteur Vallery-Radot, Presse Med., Oct. 25, 1915. The isolated germs are transferred to gelo-glucoplomb (which we cannot translate so as to give an accurate formula for the medium) and grown for 24 hours. Absence of fragmentation, with or without brown color, indicates the Eberth bacillus; Fragmentation without browning paratyphoid A; Fragmentation with browning, Paratyphoid B. The statements have been verified by agglutination tests with corresponding specific serum.

